# PPARα, a predictor of patient survival in glioma, inhibits cell growth through the E2F1/miR-19a feedback loop

**DOI:** 10.18632/oncotarget.13170

**Published:** 2016-11-07

**Authors:** Yan Shi, Tao Tao, Ning Liu, WenKang Luan, Jin Qian, Rui Li, Qi Hu, Yan Wei, Junxia Zhang, Yongping You

**Affiliations:** ^1^ Department of Neurosurgery, The First Affiliated Hospital of Nanjing Medical University, Nanjing, China; ^2^ Department of Neurosurgery, People's Hospital of Xuancheng City, Anhui, China

**Keywords:** PPARα, E2F1, miR-19a, RB, feedback loop

## Abstract

Nuclear receptors such as peroxisome proliferator-activated receptor α (PPARα) are potential therapeutic targets. In this study, we found that PPARα expression was lower in high grade gliomas and PPARα was an independent prognostic factor in GBM patients. PPARα agonism or overexpression inhibited glioma cell proliferation, invasion, and aerobic glycolysis as well as suppressed glioma growth in an orthotopic model. Bioinformatic analysis and luciferase reporter assays showed that miR-19a decreased PPARα expression. E2F1 knockdown up-regulated PPARα and inhibited cell proliferation, invasion, and aerobic glycolysis, but this activity was blocked by miR-19a. Knockdown of E2F1 decreased miR-19a by inhibiting the miR-19a promoter. Moreover, PPARα repressed E2F1 via the p21 pathwayby modulating the transcriptional complexes containing E2F1 and pRB proteins. These results suggest that the E2F1/miR19a/PPARα feedback loop is critical for glioma progression.

## INTRODUCTION

The peroxisome proliferator-activated receptor α (PPARα) is part of a family of ligand-activated nuclear steroid hormone receptors [1] act as transcription factors [2, 3]. After ligand binding, PPARα heterodimers with retinoid X receptors (RXR) and binds to specific DNA sequences to regulate expression of target genes[4]. PPARα activation is involved in glucose, lipid, and amino acid metabolism [5] and regulates a number of physiological processes including cell proliferation, apoptosis, inflammation, and oxidative stress [6, 7].

The link between PPARα and cancer was first made after PPARα agonists were shown to increase the incidence of liver tumors in rodents [8, 9]. This finding was not replicated in humans, and consequently, PPARα is not an established molecular target for cancer therapy. However, recent studies found reduced levels of PPARα in some tumor types [10–12], while PPARα agonists can suppress tumor growth [13] [14]. The PPARα agonist, fenofibrate, induces human HepG2 cell death by increasing the levels of reactive oxygen species (ROS) and depleting intracellular (GSH) [15]. PPARα activation also suppresses the metastatic potential of melanoma *in vitro* and *in vivo* [16, 17]. Nonetheless, in other cancers, including kidney [12] and hepatocellular carcinoma [18], PPARα has also been found to lead to the progression of tumor growth. Thus, the biological function of PPARα in human cancers is still controversial and its role needs further investigation.

This study explores the clinical features, biological functions and potential mechanisms of action of PPARα in glioma both *in vitro* and *in vivo*. We found that PPARα is associated with glioma grade and GBM survival. PPARα inhibits glioma cell proliferation, invasion, and aerobic glycolysis, and suppresses glioma growth in an orthotopic model via a positive feedback loop with E2F1 and miRNA-19a.

## RESULTS

### Lower PPARα expression is associated with poorer clinical outcomes in glioma patients

**Figure 1:**
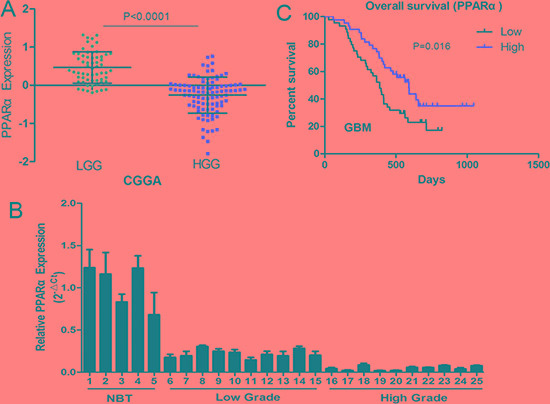
PPARα expression in glioma tissues of the CGGA glioma dataset (61 cases of low grade glioma [LGG], 97 cases of high grade glioma [HGG]) (**A**) PPARα expression was significantly lower in HGGtissues than in LGG tissues. (**B**) qRT-PCR confirmation of reduced PPARα levels in HGG tissues compared with LGG tissues and normal brain tissues. (**C**) Kaplan-Meier survival curves for PPARα expression in glioma tissues of the CGGA dataset. Low expression of PPARα confers a poor prognosis in glioma patients.

PPARα expression was analyzed in whole genome gene profiling of 158 glioma tissues based on Chinese Glioma Genome Atlas (CGGA) data. PPARα expression was significant lower in high grade gliomas (HGG) compared to low grade gliomas (LGG) (Figure 1A). Two employed two independent glioma gene expression data sets (Rembrandt data and GSE4290 data) were uesd to examine the association between PPARα expression levels and glioma grade (Figure S1A). PPARα was associated with tumor grade (*P* < 0.0001 for both Rembrandt data and GSE4290 data), which is consistent with the CGGA data. PPARα expression was measured in 5 normal brain tissues and 20 glioma tissues using qRT-PCR and a similar trend of low expression of PPARα in glioma tissues was observed (Figure 1B). GBM samples expressing lower than a median level of PPARα were associated with decreased survival relative to those with PPARα levels higher than the median (*P* = 0.016) in the CGGA data (Figure 1C). Moreover, PPARα expression was positively correlated with overall survival based on Rembrandt and GSE4290 data (Figure S1B). Overall, these data suggest that PPARα inactivation may play an important role in glioma development and survival prediction.

### PPARα is an independent prognostic factor in GBM patients

Univariate Cox regression analysis was performed using clinical and genetic variables for 89 GBM patients from the CGGA. High expression of PPARα, high Karnofsky Performance Status (KPS) score, total tumor resection, and high levels of the proliferation markers, Ki-67, PCNA, P170 and high TOPO II were all associated with overall survival, whereas gender and increasing age were not (Table 1). Interestingly, high expression of PPARα was associated with gender (*P* = 0.043), and older age at diagnosis (*P* = 0.001) (Table S1). We evaluated the factors that contribute to overall survival with a multivariate Cox proportional hazards model. PPARα expression, KPS score, and total resection all correlated independently with overall survival (HR = 0.465, *P* = 0.006; HR = 0.389, *P* = 0.002; HR = 0.503, *P* = 0.042; respectively) when considering gender, Ki-67, P170, PCNA and TOPO II expression (*P* < 0.2, univariate Cox regression analysis).

**Table 1:** Cox proportional hazard regression analyses of PPARα expression and other characteristics in relation to overall survival in GBM patients

Variable	Univariable Regression		Multivariable Regression		
	HR	*P* value	HR	*P* value
Gender (Female/ Male)	1.327	0.293		
Increasing age	1.008	0.455		
KPS score (> 80)	0.310	< 0.001	0.389	0.002
Total resection	0.560	0.028	0.503	0.042
IDH1 mutation	0.734	0.422		
MGMT promoter methylation	1.136	0.713		
High PPARα	0.532	0.016	0.465	0.006
High Ki-67	1.508	0.127	1.275	0.438
High EGFR	1.430	0.201		
High MGMT	0.905	0.966		
High PCNA	1.571	0.111	2.243	0.013
High P170	0.622	0.077	1.546	0.438
High PTEN	1.036	0.972		
High TOPO II	1.618	0.087	1.394	0.311
High GST-π	1.393	0.217		

### PPARα suppresses glioma growth *in vitro* and in an orthotopic model

**Figure 2:**
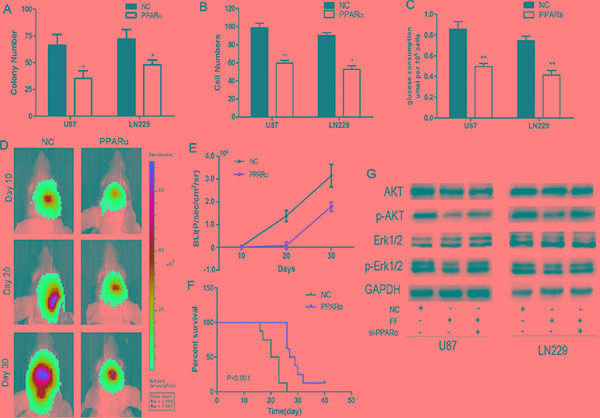
Effects of PPARα on glioma cell biology as evaluated with *in vitro* assays (**A**) Overexpression of PPARα decreased the number of colonies formed in plates. (**B**) Invasiveness of U87 and LN229 cells was attenuated by increased expression of PPARα. (**C**) Over-expression of PPARα reduced the levels of aerobic glycolysis in U87 and LN229 cells. (**D**) Representative images of mice implanted with intracranial tumors on days 10, 20, and 30. (**E**) Plot of Fluc activity by bioluminescence imaging for intracranial tumors. (**F**) Kaplan-Meier survival curves for PPARα expression in the two groups of mice. (**G**) The PPARα agonist fenofibrate interferes with the Akt and Erk1/2 signaling pathways by decreasing Akt and Erk1/2 phosphorylation. The effect of fenofibrate on Akt and Erk1/2 phosphorylation in a PPARα-dependent manner.

To determine the biological functions of PPARα, PPARα expression were transfected with PPARα-lentivirus (Figure S2A). Overexpression of PPARα in both U87 and LN229 glioma cells with low endogenous PPARα expression inhibited cell colony formation, invasion, and glucose consumption (Figure 2A–2C and Supplementary Figure S2B and S2C). We verifed the inhibitory effect of PPARa on glioma cells by adding fenofibrate to our cultures. Fenofibrate also notably suppressed glioma cell proliferation, invasion and aerobic glycolysis (Supplementary Figure S2D–S2F).

To further investigate the role of PPARα in tumor growth of glioma *in vivo*, we extended our investigation by intracranial implantation of PPARα overexpressing U87 cells in nude mice. Bioluminescence imaging showed tumor growth stasis in the overexpressing PPARα group compared with the control group and on day 10, a statistically significant difference in tumor volume emerged between the 2 groups (Figure 2D and 2E). Moreover, overexpression of PPARα markablely prolonged the survival time of glioma-bearing mice (Figure 2F).

Both Akt and Erk1/2 play a role in malignant progression, thus, we evaluated phosphorylation of these proteins after fenofibrate treatment [17]. Fenofibrate reduced Akt and Erk1/2 phosphorylation. To prove that these effects were PPARα dependent, we tranduced si-PPARα into cells before fenofibrate treatment. As shown in Figure 2G, si-PPARα abolished the role of fenofibrate indicating PPARα may affect cellular behavior in glioma by inhibiting the phosphorylation status of Akt and Erk1/2.

### E2F1/miR-19a negatively regulates PPARα in glioma cells

**Figure 3:**
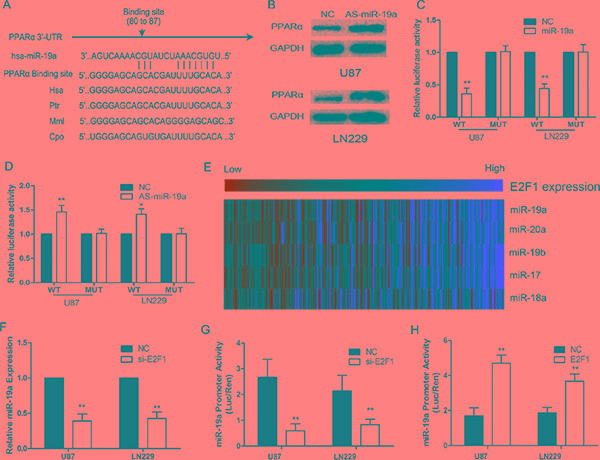
PPARα is negatively regulated by E2F1/miR-19a signaling in glioma cells (**A**) Schematic of the PPARα 3′UTR including the putative binding sites for miR-19a, as predicted by TargetScan and Pictar algorithms. (**B**) PPARα protein levels in U87 and LN229 cells at 48 h post-transfection. (**C**) MiR-19a down-regulated luciferase activity of wild-type PPARα 3′UTR expression vector, but did not reduce expression of a mutant 3′UTR. (**D**) Heatmap showing the miR-17-92 cluster (including miR-19a) positively correlated with E2F1 in 158 glioma samples. (**E**) si-E2F1 decreases the expression of miR-19a in U87 and LN229 cells. (**F**) si-E2F1 inhibits miPPR-19a activity in a luciferase assay. (**G**) Increased E2F1 expression enhances miPPR-19a activity.

We used bioinformatic analysis, TargetScan and PicTar, to investigate the upstream of PPARα in glioma cells. We identified PPARα as the potential target of miR-19a (Figure 3A). Western blot analysis showed that PPARα expression was increased in glioma cells with down-regulation of miR-19a (Figure 3B). We determined the direct interaction between miR-19a and its binding site within PPARα mRNA, usingluciferase reporter constructs containing either wild-type (pGL3-WT- PPARα-3′UTR) or mutant (pGL3-MUT- PPARα-3′UTR) PPARα 3′UTRs transfected into glioma cells. MiR-19a could influence luciferase activity of the pGL3-WT- PPARα-3′UTR plasmid in U87 and LN229 cells without significantly changing activity of the pGL3-MUT- PPARα-3′UTR plasmid (Figure 3C and 3D). These data provide evidence that miR-19a directly suppresses PPARα expression by binding to the 3′UTR of PPARα mRNA in gliomas.

The expression of miR-19a was significantly higher in HGG than LGG, and a reduction in miR-19a inhibited glioma cell proliferation, invasion and aerobic glycolysis (Supplementary Results and Figure S3). E2F transcription factors can directly bind the promoter of the miR-17-92 cluster regulating its transcription[19]. Thus, microarrays combined with Pearson correlation analysis revealed that the miR-17-92 cluster was positively correlated with E2F1 in glioma cells (Figure 3E). siRNA knockdown of E2F1 decreased miR-19a expression (Figure 3F). Further, we created a construct containing the miR-19a promoter (miPPR-19a) showed reduced activity in si-E2F1 cells (Figure 3G), while up-regulation of E2F1 using an over-expression construct notably enhanced miPPR-19a activity (Figure 3H). Taken together, these results show that E2F1 increases miR-19a expression.

**Figure 4:**
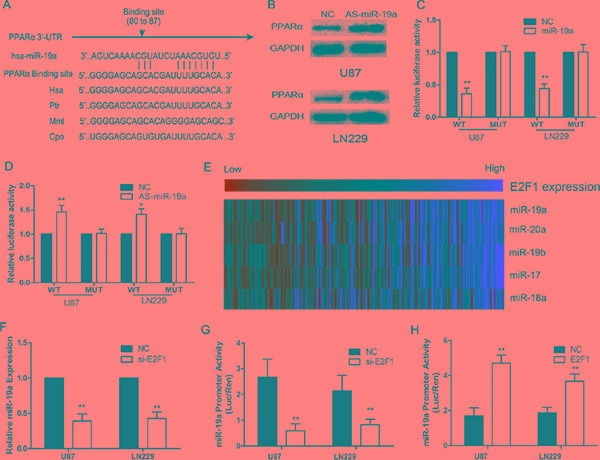
E2F1 affects the biological behavior of glioma cells, modulating PPARα in a miR-19a-dependent manner (**A**) The levels of E2F1 were analyzed in glioma tissues of the CGGA glioma datasets (61cases of LGG, 97 cases of HGG). (**B**) qRT-PCR confirmation of decreased E2F1 levels in HGG tissues compared with LGG tissues and normal brain tissues. (**C**) Kaplan-Meier survival curves for E2F1 expression in glioma tissues of the CGGA dataset. High expression of E2F1 confers a poor prognosis in glioma patients. (**D**) Decreased E2F1 suppressed the proliferation of glioma cells and its effects were blockedby miR-19a. (**E**) Decreased E2F1 suppressed the proliferation of glioma cells and its effects were blockedby miR-19a. (**F**) The invasiveness of U87 and LN229 cells was attenuated with the increased expression of E2F1. The effect of E2F1 on glioma cells is abolished by miR-19a. (**G**) Western blots identified that miR-19a could restore siE2F1 inducing PPARα expression decreased.

Next, we analyzed the E2F1 expression pattern in CGGA data and verified it in 25 tissues samples. As shown in Figure 4A and 4B, E2F1 was higher in the HGG samples than in the LGG samples. Kaplan-Meier survival curve analysis showed that high expression of E2F1 confers a poor prognosis in GBM patients (Figure 4C). si-E2F1 triggered the inhibition of glioma cell proliferation, invasion and aerobic glycolysis (Figure 4D–4F and Supplementary Figure S4A and S4B). Furthermore, increased miR-19a in E2F1-depleted cells largely blocked the effect of si-E2F1 on the suppression the malignant progression and the decreased PPARα expression in glioma cells (Figure 4G), suggesting that E2F1 enhances malignant glioma progression by decreasing PPARα expression in a miR-19a-dependent manner.

### PPARα activation feedback represses E2F1 activation by modulating transcriptional complexes formed with E2F1 and pRB proteins

**Figure 5:**
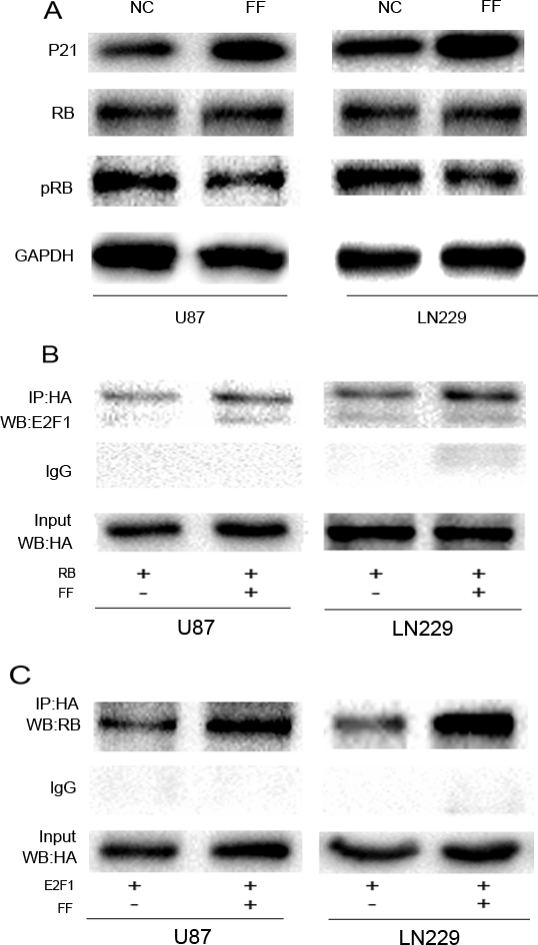
Active PPARα feedback represses E2F1 activation (**A**) PPARα activation increases p16 and p21 protein levels and decreases CDKI-mediated pRB phosphorylation. (**B**, **C**) The PPARα agonist fenofibrate promoted E2F1/RB complex formation in glioma cells.

To determine whether PPARα feedback regulates the expression or trans-activation of E2F1 through a PPARα-dependent mechanism, we analyzed the sequence of the E2F1 promoter region. There was no PPAR-response element (PPRE), suggesting that E2F1 may be modulated by PPARα via an indirect mechanism. PPARα activation increases p16 protein levels through direct DNA binding to the p16 promoter [20]. P16 protein is a tumor suppressor belongings to the super-family of cyclin-dependent kinase (CDK) inhibitors (CDKI)[21]. These proteins inhibit cell-cycle progression by preventing the association of CDKs with cyclins(CYCs), and initiate RB phosphorylation by CYC/CDK complexes [22, 23]. PPARα agonist treatmentenhanced p21 (another CDKI family member) protein levels in U87 and LN229 cells and decreased the ratio of phosphorylated/non-phosphorylated RB (Figure 5A). Because the trans-activation potential of E2F is repressed by the RB family of proteins through the formation of E2F/RB complexes [24], and RB phosphorylation can disrupt E2F/RB complexes, we hypothesized that PPARα regulates the biological function of E2F1 by interfering with E2F/RB complexes via the p21 signaling pathway. Co-immunoprecipitation (co-IP) assays showed that E2F1 coprecipitated with RB in protein lysates isolated from U87 and LN229 cells. In addition, PPARα activation resulted in increased binding of E2F1 to the RB. These results indicate that PPARα activation directly inhibits E2F1 activation by preventing the disruption of RB/E2F1 complexes, while RB binding inhibits the ability of E2F1 to form the protein-protein contacts required for activation and may serve as a trans-activator. (Figure 5B and 5C).

### Clinical significance of E2F1/ miR-19a/ PPARα feedback loop in glioma tissues

**Figure 6:**
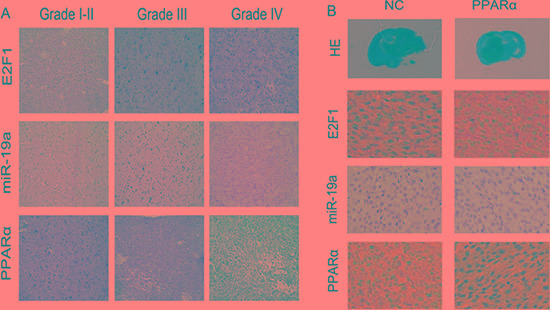
E2F1/ miR-19a/ PPARα signaling was confirmed in human glioma tissues and in nude mice orthotopic glioma model (**A**) Expression of E2F1, miR-19a and PPARα in human glioma tissues by IHC and ISH. (**B**) E2F1/ miR-19a/ PPARα feedback loop was confirmed in nude mouse orthotopic glioma model.

Immunohistochemistry and *in situ* hybridization analysis revealed that E2F1 and miR-19a were increased in HGG in comparison with LGG, whereas PPARα was decreased in HGG (Figure 6A and Supplementary Table S2). E2F1 and miR-19a expression were markedly decreased following up-regulation of PPARα in a nude mouse glioma xenograft model (Figure 6B). In glioma tissues, pearson correlation showed a significant positive correlation between E2F1 and miR-19a levels (R = 0.66, *P* < 0.01). An inverse correlation was detected between miR-19a and PPARα (R = −0.62, *P* < 0.01), as well as PPARα and E2F1 (R = −0.68, *P* < 0.01).

## DISCUSSION

Nuclear receptors are promising novel therapeutic targets for cancer treatments. Peroxisome proliferator-activated receptors (PPARs), which belong to the nuclear hormone receptor super family and are ligand-activated transcription factors [11], are among these targets. PPARα agonists suppress human colorectal carcinoma cell growth [25]. The PPARα agonist fenofibrate suppresses tumor growth and angiogenesis by reducing endothelial cell proliferation and VEGF production and increasing endostatin [26]. In the current study, PPARα has been identified as a critical marker not only of tumor grade but also for prognosis in glioma. PPARα inhibited the malignant progression of glioma *in vivo* and *in vitro*. Our results show that PPARα is an important factor for malignant progression survival in glioma patients and exhibits antitumor effects by reducing cell proliferation, invasion and aerobic glycolysis.

The E2F family has been verified as a promising target gene related to the G1/S transition. E2F controls gene expression at G1/S by activating genes that encode DNA replication proteins, enzymes that are responsible for deoxynucleotide biosynthesis, proteins that assemble to form functional origin complexes and kinases that are involved in the activation of initiation. E2F1 is a downstream regulator of the Rb pathway, which is capable of inducing cell proliferation and cell cycle progression [27–29]. Interestingly, E2F1 has multiple functions that could be considered to be either suppressing or promoting tumor development. Some previous studies support the role of E2F1 as a tumour suppressor rather than an oncogene [30, 31]. To date, the dysregulation, function, upstream regulators, and downstream effectors of E2F1 remain unclear. Recently, a cluster of miRNAs determining the regulation of E2F1 expression has been discovered. For example, members of the miR-17-92 cluster such as miR-20a and miR-17-5p inhibited E2F1 expression at the post-transcriptional level. As a transcription factor, E2F1 increased the expression of microRNAs within the miR-17–92 cluster[32]. However, other studies, [33] including the present study, have identified E2F1 oncogenic functions in gliomas.

In this study, we used bioinformatic and integrative analytical approaches to detect the underlying function and molecular mechanism of PPARα in glioma. The depletion of E2F1/miR-19a signaling inhibited glioma cell proliferation, invasion and aerobic glycolysis accompanying the downregulation of PPARα. MiR-19a was an important mediator between E2F1 and PPARα through qRT-PCR, Western blot, and luciferase reporter assays. Increased miR-19a expression largely blocked the effect of si-E2F1 on PPARα expression. Interestingly, E2F1 was regulated by PPARα through the p21 pathway, which can disrupt E2F/RB complexes by increasing RB phosphorylation and activating E2F1. However, the mechanism of how p21 is upregulated following PPARα activation remains unclear. In human glioma samples, Pearson's correlations showed a significantly positive correlation between between E2F1 and miR-19a. An inverse correlation was detected between E2F1 and PPARα levels, as well as miR-19a and PPARα. To our knowledge, we are the first to propose that the E2F1/miR-19a/PPARα feedback loop is a key regulatory element in glioma. Further investigation into this feedback loop in glioma could lead to the identification of new therapeutic targets.

## MATERIALS AND METHODS

### Tissue samples and microarray analysis

Information on the tissue samples is described in Supplementary Materials and Methods. Microarray analysis was performed as previously described [34]. Glioma gene expression datasets are deposited at the Repository of Molecular Brain Neoplasia Data (REMBRANDT; http://caintegrator.nci.nih.gov/rembrandt/) and the Gene Expression Omnibus Web site (http://www.ncbi.nlm.nih.gov/geo/, accession nos. GSE4290).

### Cell lines and chemicals

The human U87 and LN229 glioma cell lines used here were purchased from the Chinese Academy of Sciences Cell Bank. Both cells lines were cultured in Dulbecco's modified Eagle's medium (DMEM) (Gibco, USA) supplemented with 10% fetal bovine serum and maintained at 37°C in a 5% CO_2_ incubator. In some experiments, PPARα was activated by stimulating serum-starved cells with 100 μM of fenofibrate (Gibco, USA).

### Gene knockdown and overexpression

The 2′-O-methy1 (2′ – OMe-) oligonucleotides were chemically synthesized by GenePharma (Shanghai, China). The sequences are as follows: 2′-OMe-hsa-miR-19amimics (miR-19a), 5′-UGU GCA AAU CUA UGC AAA ACU GA-3′; E2F1 small interfering RNA (siRNA), 5′-CUG CAG AGC AGA UGG UUA UTT-3′; PPARα- siRNA, 5′-UCA CGG AGC UCA CAG AAU UUU-3′ and 3′-AAU UCU GUG AGC UCC GUG AUU-5′; scrambled siRNA (negative control), 5′-UUC UCC GAA CGU GUC ACG UTT-3′; 2′-OMe-hsa-miR-19a inhibitor (AS-miR-19a), 5′-UCA GUU UUG CAU AGA UUU GCA CA-3′; and mircoRNA inhibitor negative control, 5′-CAG UAC UUU UGU GUA GUA CAA-3′. Oligonucleotides (20 μM) were transfected into glioma cells using Lipofectamine 2000 (Invitrogen). The lentiviral vector Ubi-MCS-3FLAG-SV40-EGFP, containing the ORF of PPARα (PPARα), was generated by GeneChem Inc. The empty lentiviral expression vector was used as a negative control. Oligonucleotides were complexed with Lipofectamine 2000 through OPTI-MEM (Invitrogen), subsequently added to glioma cells at a final concentration of 50 nmol/L, and left to incubate. Then 8 hours later, the media was changed to DMEM. The E2F1-pSG5L-HA plasmid (Addgene plasmid 10736) and RB-pSG5L-HA (Addgene plasmid 10720) plasmid were obtained from Addgene (USA).

### Cell counting kit-8 (CCK8) assay and colony formation assay

CCK8 assay: The cells were plated in 96-well plates in medium containing 10 % FBS at approximately 5,000 cells per well 24 h after transfection. Subsequently, oligonucleotides were transfected or fenofibrate (Sigma, USA) was added into the cells. After 24 h, 48 h, 72 h, and 96 h, 10 μl of CCK8 (Beyotime, China) was added into each well and then the cells were incubated at 37°C for an additional 3 h. The optical density was measured at 450 nm wave length. Colony formation assay: The cells were plated in triplicate in 60 mm plates in the presence of blank lentiviral vector or lentiviral- PPARα for 14 days until the colonies were sufficiently large for visualization. Colonies were then fixed in methanol for 10 min and stained with 0.1% crystal violet for 10 min.

### *In vitro* invasion assay

The cells were transfected with oligonucleotides and incubated at 37°C for 48 hours, and then we transfer the cells were transferred to the top chambers Matrigel-coated invasion chambers (24-well insert, 8-μm pore size, BD Biosciences, San Jose, USA) in a serum-free DMEM and the medium containing 10% fetal bovine serum was added to the lower chamber to function as the chemoattractant. After incubating for 24 h, the remaining cells in the chambers were removed using cotton swabs and the invading cells on the lower surface of the chambers were fixed with 95% ethanol, stained with 0.1% crystal violet, and photographed (×200) in three independent fields for each well. Tests were repeated via three independent experiments.

### Glucose assay

Cells were seeded into 6-well plates at 3 × 10^5^ cells per well in 2 ml of supplemented DMEM. Subsequently, oligonucleotides were transfected or fenofibrate was added to the cells. After 48 h, 2 μl of supplemented DMEM was added into a series of well on a 96-well plate. The glucose assay was described in Supplementary Materials and Methods.

### RNA extraction and quantitative RT -PCR

RNA was extracted from the cells after transfection or from tissues using TRIzol (Invitrogen). The E2F1 and PPARα (qRT-PCR) reactions were performed using Fermentas reverse transcription reagents and SYBR Green PCR Master Mix (Applied Biosystems) according to the manufacturer's protocols. GAPDH was used for normalization. MiR-19a qRT-PCR reactions were performed using TaqMan miRNA assays (Applied Biosystems). U6 was used for normalization. And analysis was performed using the 2^–ΔCt^ or 2^–ΔΔCt^ method. Each experiment was performed in triplicate.

### Co- immunoprecipitation (co-IP)

For Co-IP, U87 and LN229 cells seeded in 100 mm plates were transfected with E2F1-pSG5L-HA (Addgene plasmid 10736) or RB-pSG5L-HA (Addgene plasmid 10720). Cells were treated with or without 100 μM Fenofibrate 24 h post-transfection, and lysed for 30 min in lysis buffer 48 h post-transfection. Lysate supernatants prepared as above were incubated with 20 μl mouse anti-HA antibody and washed protein A/G agarose beads (Santa Cruz, USA) overnight at 4°C under continuous agitation. The beads were subsequently washed four times with the lysis buffer. Bound proteins were eluted with SDS loading buffer by boiling for 5 min and were subjected to western blot assay.

### Western blotting

Western bloting was performed as previously described [35]. Immunoblots were performed using appro-priate primary antibodies: E2F1 (1:500, Abcam, UK); PPAR alpha (1:500, Abcam, UK), phospho-Akt, Akt, phospho-extracellular signal–regulated kinase (Erk) 1/2 and anti-Erk1/2 (all from Santa Cruz, USA); and phosphor-RB, RB, p16, p21 (all from CST, USA), GAPDH (1:1000, CST, USA).

### Luciferase reporter assay

The 3′-UTR of PPARα containing the putative miR-19a binding sequences was cloned into a firefly luciferase reporter construct. The 3′-UTR of PPARα without the putative miR-675 binding sequences was used as mutated controls (Invitrogen). Luciferase activity was measured using the Dual-Luciferase Reporter Assay System (Promega, USA). MiR-19a promoter-containing (miPPR-19a-containing) pGL3-Basic plasmids and mutated plasmids were constructed (SunShineBio Inc), as described previously [32]. Luciferase activity was measured using the Dual-Luciferase Reporter Assay System. (Promega, USA).

### Xenograft tumor assay

Bagg albino (BALB) nude mice at 4 weeks of age were purchased from the Animal Center of the Cancer Institute at the Chinese Academy of Medical Science. The guidelines for animal welfare were approved by the Ethics Committee on Animal Research of Tianjin Medical University which was performed as previously described [36]. To establish intracranial gliomas, 0.5 × 10^5^ U87 cells were transduced with PPARα overexpression luciferase lentivirus and then implanted stereotactically. Mice were imaged for Fluc activity using bioluminescence imaging on days 10, 20, and 30 [36, 37]. All mice were divided randomly into two groups: a control group and a PPARα overexpression group.

### Immunohistochemistry staining and *in situ* hybridization

Immunohistochemistry staining and *in situ* hybridization is described in Supplementary Materials and Methods

### Statistical analysis

All experiments were performed three times. Data were analyzed using SPSS 10.0. Statistical evaluation of the data was performed using one-way analysis of variance (ANOVA) for 3-group comparisons and *t*-tests for 2-group comparisons. The Pearson correlation analysis was performed using Matlab software. *P* < 0.05 was considered statistically significant.

## SUPPLEMENTARY MATERIALS FIGURES AND TABLES


