# Supplementary material for: PPARα, a predictor of patient survival in glioma, inhibits cell growth through the E2F1/miR-19a feedback loop
Source: Oncotarget. 2016 Nov 7;7(51):84623–33. doi: 10.18632/oncotarget.13170 (PMC5356686; doi:10.18632/oncotarget.13170)
Supplement: Supplementary file 1 [file oncotarget-07-84623-s001.pdf]

PPARα, a predictor of patient survival in glioma, inhibits cell growth through the E2F1/miR-19a feedback loop

Supplementary Materials

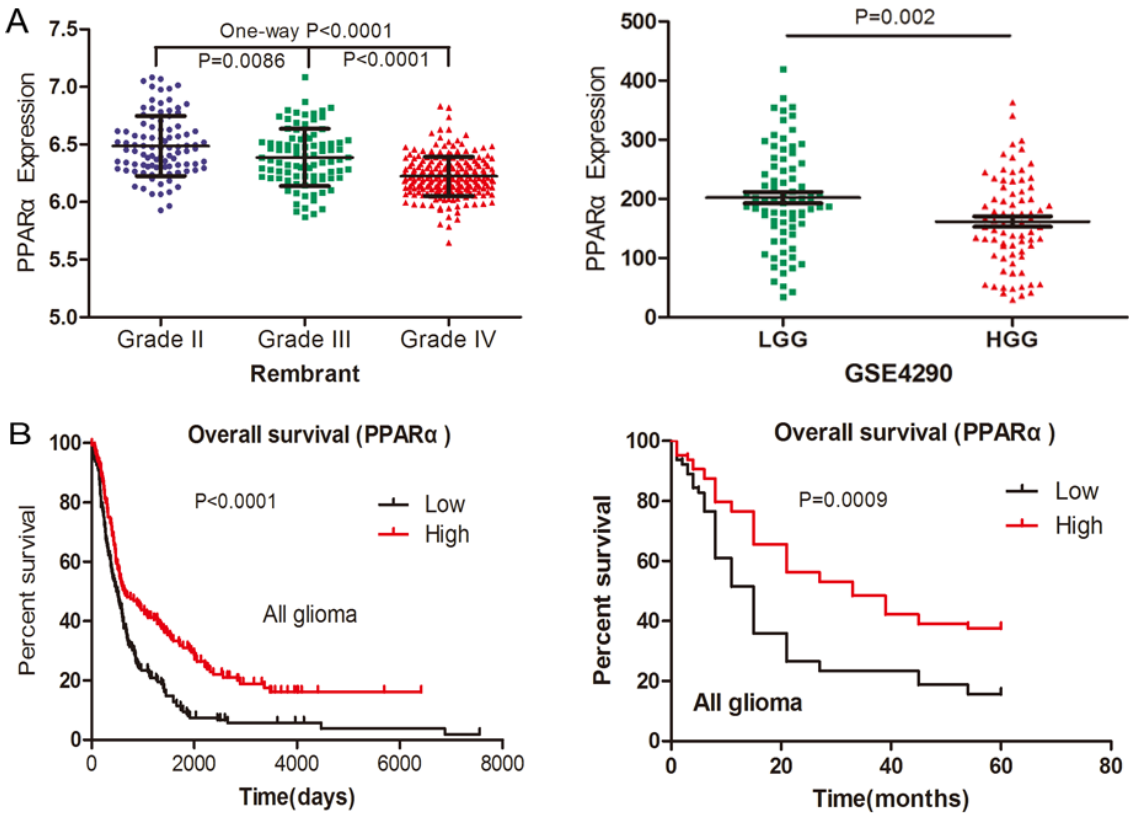

Supplementary Figure S1: The expression of PPARα in gliomas and its association with survival in glioma patients. (**A**) The levels of PPARα were analyzed in glioma tissues of the Rembrandt and GSE4290 glioma datasets. (**B**) Kaplan-Meier survival curves according to the expression of PPARα in the Rembrandt and GSE4290 glioma datasets. Low expression of PPARα confers a poor prognosis in glioma patients.

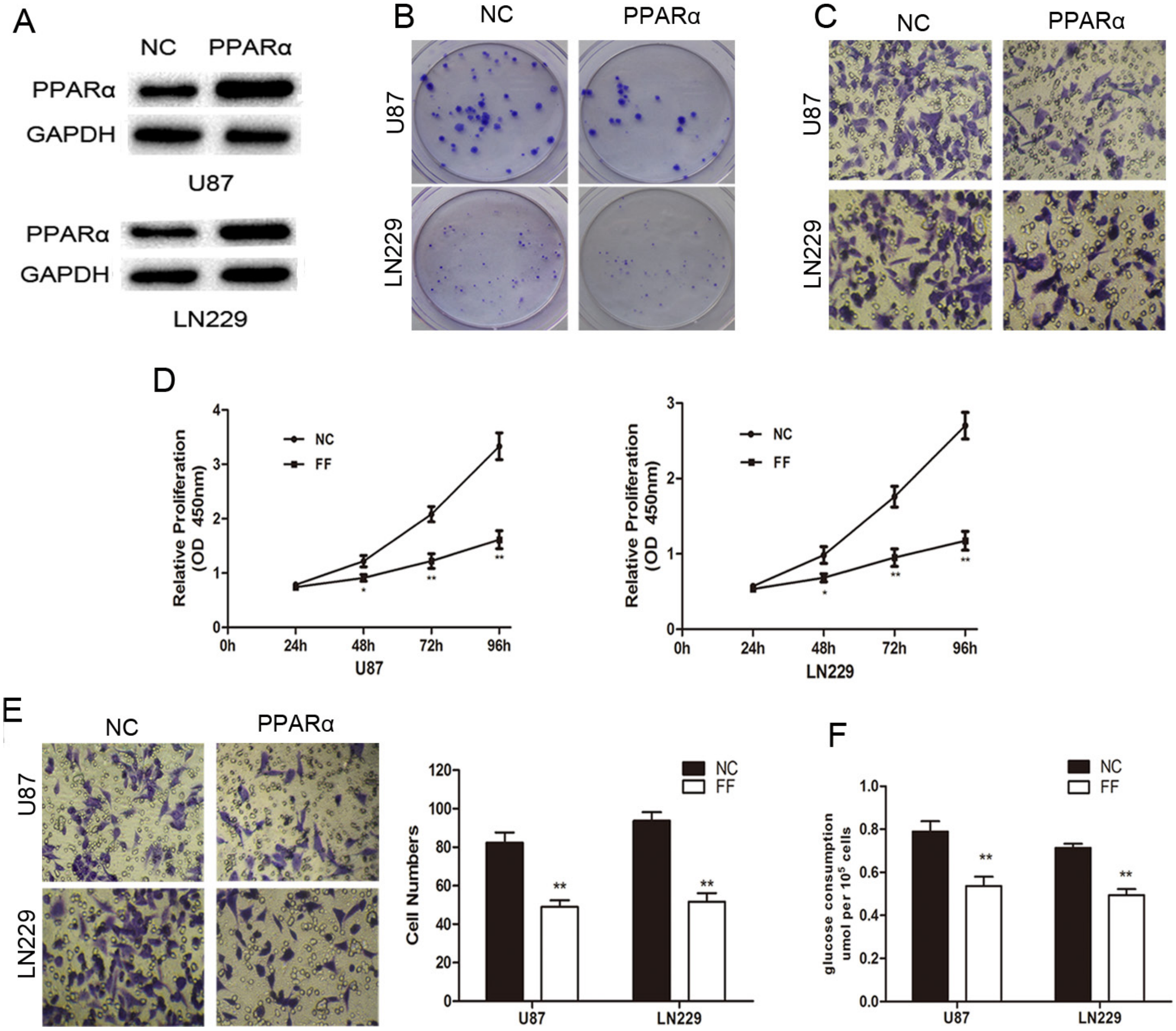

Supplementary Figure S2: Effect of PPARα on glioma cell biology evaluated using in vitro assays. (**A**) PPARα was up-regulated by lentiviral- PPARα. (**B**) Overexpression of PPARα decreases the number of colonies formed in plates. (**C**) The invasiveness of U87 and LN229 cells was attenuated with the increased expression of PPARα. (**D**) PPARα activation reduced the proliferation of glioma cell lines. (**E**) PPARα activation inhibited the invasiveness of U87 and LN229 cells. (**F**) The aerobic glycolysis of glioma cells were inhibited when PPARα activation by fenofibrate.

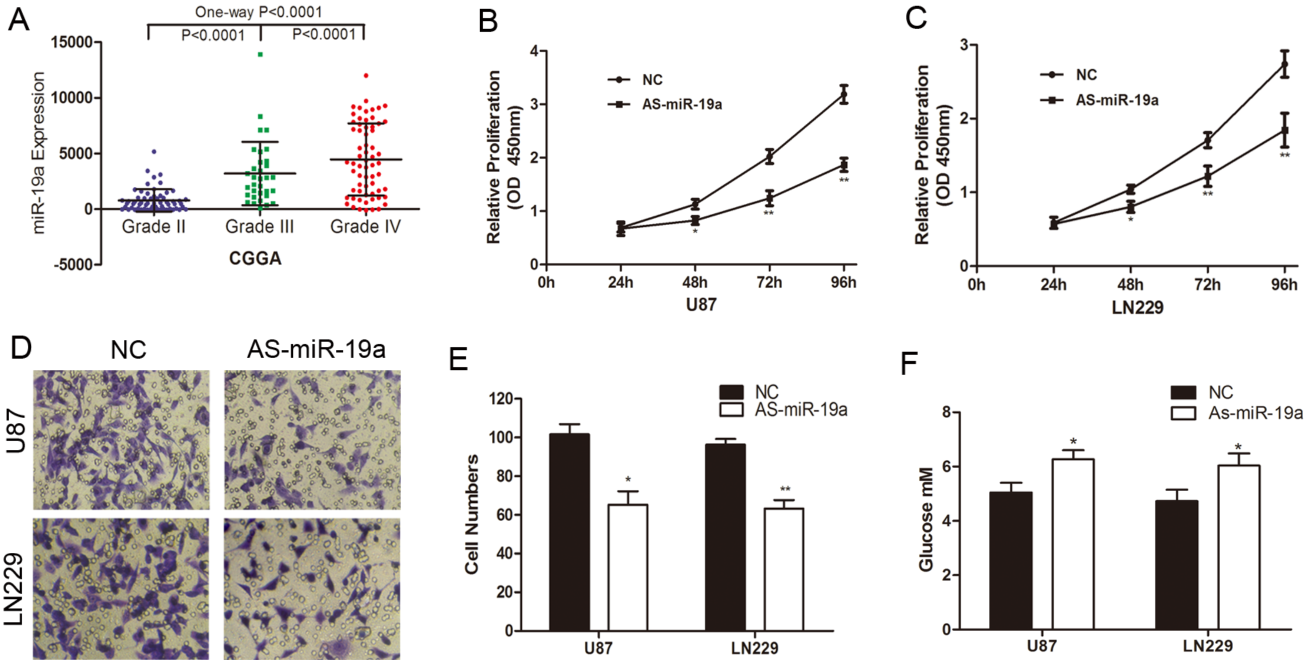

Supplementary Figure S3: MiR-19a expression and funtion in glioma. (**A**) MiR-19a levels were analyzed in glioma tissues of the CGGA glioma datasets (61cases of grade II, 33 cases of grade III and 64 cases of grade IV). (**B** and **C**) AS-miR-19a reduced the proliferation of U87 and LN229 cells. (**D** and **E**) AS-miR-19a inhibited the invasiveness of U87 and LN229 cells. (**F**) Over-expression of miR-19a reduced the levels of aerobic glycolysis inU87 and LN229 cells.

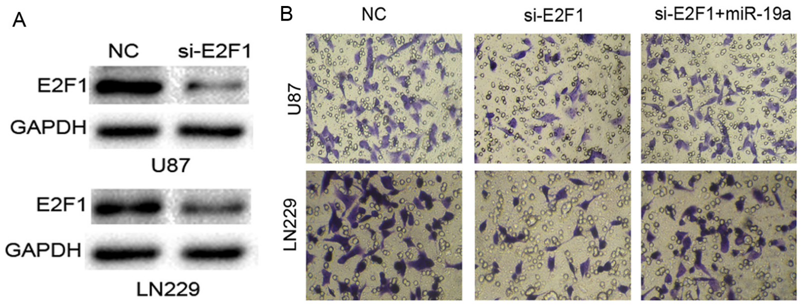

Supplementary Figure S4: E2F1 affects the biological behavior of glioma cells. (A)Expression of E2F1 was completely knocked down by siRNA. (B) Decreased E2F1 suppressed the invasion of glioma cells and its effects were blocked by miR-19a in glioma cell.

Supplementary Table S1: Clinical and molecular pathology features of GBM samples according to PPARα expression

	Low	High	*P* value
Gender (Female/ Male)	14/31	23/21	0.043
Age at diagnosis (Year)	50.0 ± 12.4	41.1 ± 11.6	0.001
Overall survival	392.3 ± 206.2	504.0 ± 219.6	0.017
KPS score (≥ 80/< 80)	28/17	31/13	0.411
Resection (Subtotal/Total)	20/25	21/23	0.756
IDH1 mutation (No mutation/ Mutation)	37/5	26/8	0.181
MGMT promoter methylation (Unmethylated/Methylated)	24/9	16/8	0.621
MGMT (Low/ High)	17/27	10/33	0.121
Ki-67 (Low/ High)	23/21	20/23	0.591
EGFR (Low/ High)	15/29	21/22	0.163
P170 (Low/ High)	20/24	19/24	0.905
PTEN (Low/ High)	1/43	1/42	0.987
PCNA (Low/ High)	30/14	34/9	0.250
TOPO II (Low/ High)	18/26	17/26	0.896
GST-π (Low/ High)	23/21	24/19	0.740

Supplementary Table S2: E2F1, miR-19a and PPARα expression in human glioma tissues

Tissue	Number	The expression of E2F1					The expression of miR-19a					The expression of PPARα				
		−	+	++	+++	Positive Rate(%)	−	+	++	+++	Positive Rate(%)	−	+	++	+++	Positive Rate(%)
**I–II**	15	8	3	4	0	47	9	2	3	1	40	5	2	5	3	67
**III**	15	4	3	5	3	73	5	4	4	2	67	9	3	2	1	40
**IV**	20	4	2	10	4	80	6	2	5	7	70	14	2	2	2	30

negative expression (−),weak expression (+), moderate expression (++),strong expression (+++).

MATERIALS AND METHODS

Tissue samples

All human glioma samples and normal brain tissue were obtained from the patients who diagnosed with WHO (World Health Organization, 2007) grade II–IV glioma. They underwent surgical resection at Department of Neurosurgery of the First Affiliated Hospital of Nanjing Medical University. After collection, every glioma tissue was immediately frozen in liquid nitrogen. 25 specimens in liquid nitrogen were collected, including 3 normal brain tissues and 12 human glioma tissues for qRT-PCR. Fifty glioma tissue samples (15 grade I–II, 15 grade III and 20 grade IV) for immunohistochemical staining and in situ hybridization were from paraffin imbedding tissues. 158 glioma data with mRNA and miRNA expression microarray were downloaded from Chinese Glioma Genome Atlas (CGGA) data portal (http://www.cgga.org.cn/portal.php). The samples comprised 48 astrocytomas (A, WHO Grade II), 13 oligodendrogliomas (O, WHO Grade II), 8 anaplastic astrocytomas (AA, WHO Grade III), 10 anaplastic oligodendrogliomas (AO, WHO Grade III), 15 anaplastic oligoastrocytomas (AOA, WHO Grade III) and 64 GBM (WHO Grade IV). High grade glioma (HGG) including AA, AO, AOA and GBM. Low grade glioma (LGG), including O and A.

In situ hybridization

Paraffin-embedded glioma tissue and xenograft tumor sections were incubated with 1:200-diluted primary antibodies against E2F1 and PPARα overnight at 4°C,followed by incubated with a biotinylated secondary antibody (1:200 dilution, Gene Tech, China) at room temperature for 1 h. Subsequently, the sections were incubated with ABC-peroxidase for 1 h, washed with PBS and stained with diaminobenzidine for 5min, counterstained with hematoxylin (Gene Tech, China). Ten randomly selected visual fields per section were examined by light microscope to evaluate the E2F1 and PPARα expression. Using antisense locked nucleic acid (LNA)-modified probes (Boster, Wuhan, China), in situ hybridization was performed. Oligonucleotide sequences were: LNA-miR-19a, 5′-TCAGT TTTGC ATAGA TTTGC ACA-3′. Sections with no labeling or with fewer than 5% labeled cells were scored as 0. Sections with 5%–30% of cells labeled were scored as 1, with 31%–70% of cells labeled as 2, and with labeling of ≥ 71% as 3, photographed (× 200). The staining intensity was scored similarly, with 0 used for negative staining, 1 for weakly positive, 2 for moderately positive, and 3 for strongly positive. The scores for the percentage of positive tumor cells and for the staining intensity were added to generate an immunoreactive score for each specimen. The product of the quantity and intensity scores were calculated such that a final score of 0-1 indicated negative expression (−), 2-3 indicated weak expression (+), 4-5 indicated moderate expression (++), and 6 indicated strong expression (+++). Each sample was examined separately and scored by 2 pathologists. Cases with discrepancies in the scores were discussed to reach a consensus.

Glucose assay

We use Glucose Assay Kit (Biovision, USA) to test the Glucose concentrations of the culture medium. After the cells were transfected for 48 h, 2 μl of medium was added into a series of well on a 96-well plate. The standard solution was diluted to 1 nmol/μl by using assay buffer. Subsequently, 0, 2, 4, 6, 8, 10 μl of standard solution were added into each well individually, adjust volume to 50 μl/well with assay buffer to generate 0, 2, 4, 6, 8, 10 nmol/well of the glucose standard, and then adjust the sample volume to 50 μl/well with assay buffer. Finally, 50 μl of the reaction mix were added to each well and incubate the reaction for 30 minutes at 37°C. The optical density was measured at 570 nm wavelength. Apply the sample readings to the standard curve. The glucose concentrations of the test samples can then be calculated.

RESULTS

MiRNA-19a expression and function in gliomas

We analyzed miR-19a expression level in 158 glioma tissues in CGGA data. One-way ANOVA analysis showed that miR-19a expression siginificantly increased in HGG than LGG (Supplementary Figure S3A). To explore the effect of miR-19a on glioma cells, in vitro CCK-8 assay, transwell assay and glucose assay were employed. These results, were similar to PPARα activation and over-expression of PPARα, showed that decreased miR-19a expression significantly suppressed proliferation (Supplementary Fig. S3B and C), invasion (Supplementary Figure S3D and E) and aerobic glycolysis (Supplementary Figure S3F) of U87 and LN229 cells. Our study indicates that miR-19a is an Oncogenic miRNA and AS-miR-19a suppresses malignant progression of glioma cells accompanying PPARα up-regulation.
